# DDT and pyrethroid resistance in *Anopheles arabiensis* from South Africa

**DOI:** 10.1186/1756-3305-6-229

**Published:** 2013-08-08

**Authors:** Luisa Nardini, Riann N Christian, Nanette Coetzer, Lizette L Koekemoer

**Affiliations:** 1 Division of the National Health Laboratory Services, Vector Control Reference Laboratory, Centre for Opportunistic, Tropical and Hospital Infections, National Institute for Communicable Diseases, Private Bag X4, Johannesburg 2131 Sandringham, South Africa; 2 WITS Research Institute for Malaria, School of Pathology, Faculty of Health Sciences, University of the Witwatersrand, Johannesburg, South Africa; 3 Bioinformatics and Computational Biology Unit, Department of Biochemistry, University of Pretoria, Pretoria, South Africa

**Keywords:** South Africa, Insecticide resistance, Cytochrome P450, Glutathione S-transferase, Permethrin, Deltamethrin, Microarrays

## Abstract

**Background:**

Pyrethroid resistance has been well documented in *Anopheles arabiensis*, one of the major African malaria vectors, and the predominant malaria vector in South Africa.

**Methods:**

In this study, the genetic basis of pyrethroid resistance in a selected laboratory strain of *An. arabiensis* from South Africa was investigated using a custom-made microarray, known as the *An. gambiae* detoxification chip.

**Results:**

A large number of P450 genes were over-transcribed, as well as a suite of redox genes and glutathione S-transferases. The five genes that showed the highest level of gene transcription when compared with an insecticide susceptible strain were: *CYP6AG2*, *CYPZ1*, *TPX2*, *CYPZ2* and *CYP6P1*.

**Conclusions:**

Permethrin resistance in South African *An. arabiensis* is associated with increased transcription of multiple genes, and a large proportion of these genes were also previously recorded as over-transcribed in another *An. arabiensis* strain selected for resistance to DDT with cross-resistance to deltamethrin. The deltamethrin resistance developed *de novo* in the DDT-selected strain and is most likely due to increased transcription of those genes associated with DDT resistance. However, of particular interest was the fact that the strain selected for resistance to pyrethroids did not develop *de novo* resistance to DDT. These differences are compared and discussed.

## Background

Pyrethroids are the most commonly used insecticides for malaria vector control. They are most often used for indoor residual spraying, and are the only class of insecticide approved for treatment of bednets due to their low levels of mammalian toxicity [1-3]. However, pyrethroid resistance in African malaria vectors, including *Anopheles arabiensis*, is well documented and has been reported in many African countries.

The Global Plan for Insecticide Resistance Management in Malaria Vectors (GPIRM) [1] provides a generic guideline for managing insecticide resistance where it occurs. Strategies for preventing and managing insecticide resistance are imperative to ensure that the limited number of insecticides available for vector control are protected. As part of this strategy, insecticide resistance should be monitored through routine surveillance and the mechanisms of insecticide resistance should be characterised where possible [1]. Information on insecticide resistance mechanisms in malaria vectors can be obtained using several techniques that vary in sophistication and cost. The more costly methodologies are not feasible in many African countries where the disease burden is the greatest.

Resistance to pyrethroids is mainly due to two mechanisms; enhanced enzyme detoxification or reduced target site sensitivity. Cytochrome oxidases (P450s), glutathione S-transferases (GSTs) and esterases are generally associated with enzymatic detoxification of insecticides, while mutations in the voltage-gated sodium channel (commonly referred to as knockdown resistance or “*kdr*”) effectively decrease target site sensitivity [4,5]. The occurrence of *kdr* mutations is easily detected through the use of a polymerase chain reaction (PCR) assay [5] but this mechanism is not clearly correlated with all instances of pyrethroid resistance, especially deltamethrin resistance [6]. Identifying metabolic based resistance is more challenging and costly, especially when using microarrays [7]. The use of microarray technology is particularly helpful as it allows for rapid analysis of numerous genes simultaneously, and as such provides substantial genetic information relating to a particular phenotype.

Genes associated with pyrethroid resistance include the esterase, GST and P450 super families [8-13]. Metabolic detoxification of pyrethroids has been demonstrated in *An. gambiae*[12,14,15], *An. arabiensis*[8,10,16] and *An. funestus* (reviewed by [17]). *Anopheles arabiensis* is a major vector of malaria in Africa including South Africa [18,19]. Historically, this species was considered to be susceptible to all classes of insecticide. In 1996 an extensive survey of insecticide susceptibility in *An. arabiensis* was conducted in three areas in South Africa (KwaZulu-Natal [KZN], Limpopo Province and the Kruger National Park) [20]. Bioassays revealed that samples of *An. arabiensis* from all three areas were susceptible to DDT and deltamethrin [20].

Current malaria vector control in South Africa is based on a mosaic approach using DDT and pyrethroids [19]. These interventions are the mainstay of mosquito vector control in South Africa even though *An. arabiensis* is both exophilic and endophilic. In 2003, *An. arabiensis* sampled from two areas in KZN were found to be resistant to DDT, but susceptible to deltamethrin [21].

Subsequent studies were initiated to investigate the molecular basis for the reported DDT resistance in KZN *An. arabiensis*. The use of laboratory strains to study resistance mechanisms carries the advantage of excluding factors that can confound the analysis (e.g. exposure to agricultural insecticides, effect of temperature, larval diet, etc). Laboratory based DDT selection pressure was exerted on this population over a period of several years and resulted in the fixation of the L1014F *kdr* mutation [8]. In addition to target site resistance, metabolic detoxification was also identified and numerous gene transcripts were over-expressed and associated with the resistant phenotype [8].

Three years after the discovery of DDT resistance, pyrethroid resistance was also reported in wild caught *An. arabiensis* from KZN [16]. This population was colonised in 2005 and selected for resistance to permethrin in order to characterise the resistance mechanisms involved. Pyrethroid resistance in this population (named KWAG-perm) was primarily associated with increased activity of monooxygenases (P450’s) [16]. Analysis of six specific P450 genes showed increased transcription for three genes in association with the pyrethroid resistant phenotype [22].

The aim of this study was to evaluate the levels of transcription of detoxification enzymes associated with the South African permethrin resistant strain (KWAG-perm) using a high throughput microarray analysis to supplement the information obtained by Munhenga and Koekemoer [22]. In addition to this, the transcript profiles between the pyrethroid-selected (KWAG-perm, permethrin resistant) and DDT-selected (MBN-DDT, DDT and pyrethroid resistance) strains were compared to identify both strain specific transcripts as well as transcripts shared between these two strains. The implications of these results for malaria control are discussed.

## Methods

### Mosquito strains

#### Pyrethroid resistant strain (KWAG-perm)

The *An. arabiensis* permethrin resistant strain, KWAG-perm, and the equivalent susceptible strain, KWAG-base, were used for this experiment. KWAG-base originates from Mamfene in KwaZulu-Natal (KZN) province, South Africa. This population was characterised as permethrin resistant at the time of colonisation [16]. However, in the absence of selection by permethrin exposure, the laboratory strain reverted back to being susceptible (after a period of one year). When permethrin selection was reintroduced, resistance rapidly returned (within four generations) and this strain was named KWAG-perm (Table 1) [16]. Mosquitoes were reared under standard insectary conditions (26°C ± 2°C, 70-80% relative humidity, and 12:12 light:dark cycle).

**Table 1:** **Summary of the *An. arabiensis *strains used in the study**

**Strain**	**Selected on:**	**Resistant to:**
KWAG-base	No selection	-
KWAG-perm	Permethrin (0.75%)	Permethrin
MBN-base	No selection	DDT (low-level)
MBN-DDT	DDT (4%)	DDT, deltamethrin and permethrin (cross-resistance)

#### DDT resistant strain (MBN-DDT)

Details on this strain can be found in Nardini *et al.*[8]. Briefly, MBN-base was colonised from Mamfene, KZN, and a sub-strain was selected for resistance to DDT. The selected strain, MBN-DDT, is resistant to DDT (0% mortality) as well as permethrin (4%), deltamethrin (34%), bendiocarb (77.5%) and propoxur (65.3%) through cross-resistance mechanisms (Table 1). The strain is homozygous for the L1014F *kdr* mutation which is mainly responsible for the observed DDT and permethrin resistance [8]. The deltamethrin resistance is mainly due to enhanced monooxygenase enzymatic detoxification [8].

### RNA extractions and preparation of amplified mRNA

#### Pyrethroid resistant strain (KWAG-perm)

RNA extractions were carried out according to Nardini *et al.*[8]. Briefly, female mosquitoes were maintained on a 10% sugar solution and RNA was extracted from 15 mosquitoes three days after emergence, representing one biological repeat. RNA was extracted for three biological repeats using the PicoPure® RNA Isolation Kit (Arcturus) according to supplied methodology (with the inclusion of a DNase treatment, as described in the user manual). RNA was quantified using a NanoDrop and quality was assessed using an Agilent 2100 Bioanalyzer. Extracted RNA was stored at -70°C. In order to obtain larger quantities of mRNA for microarrays, the RiboAmp™ RNA Amplification Kit (Arcturus) was used. Amplified mRNA was quantified using a NanoDrop.

### Preparation of microarrays and data analyses

#### Pyrethroid resistant strain (KWAG-perm)

Three independent biological repeats were used, and for each biological repeat, two technical repeats were performed that included dye swaps in order to compensate for dye bias. Microarrays were prepared as described in Nardini *et al.*[8].

The arrays were scanned using the Genepix 4000B scanner (Molecular Devices, USA). The photomultiplier tube (PMT) values were adjusted to give a pixel ratio of approximately 1. Spot quality and background intensities were examined and corrected using Genepix Pro 6.0 software (Axon Instruments, USA). Saturated features were excluded from the analysis.

Gene expression data were analysed using Limma version 2.12.0 (Bioconductor) [23] in R, version 2.8.0 (http://cran.r-project.org/bin/windows/base/old/2.8.0/). Raw intensity values for each spot were calculated, and then background corrected by the method “normexp” with an offset of 50 [24]. The corrected intensity values were transformed to log-ratios and then normalised. Composite Loess was used for within array normalisation. In this method, control spots and features, per sub-array, are used for producing non-linear, best-fit lines [25]. The use of control spots ensures that the resulting best-fit line is not biased by differential expression of genes. Conversely, the use of all genes for normalisation improves stability with respect to the number of spots and, most importantly, provides flexibility in terms of print-tip group trends that might be observed where sub-array Loess curves are used [26]. Print-tip peculiarities were present in some slides, hence the choice of the normalisation method. The “Aquantile” method was used for between array normalisation. Genes with adjusted *p*-values ≤ 0.05 and fold-changes (FCs) ≥ 2.0 were considered to be statistically significant. These data have been deposited into Vectorbase (https://www.vectorbase.org).

### Quantitative real-time PCR (qPCR)

#### Pyrethroid resistant strain (KWAG-perm)

Real-time PCR was carried out in order to validate the outcome of the microarray experiments. RNA was extracted from 15 three day old *An. arabiensis* females (represents one biological repeat, and three biological repeats were prepared) that had been supplied with 10% sugar solution. TRI-Reagent® solution (Sigma-Aldrich) was used according to the supplied methodology with a DNase treatment included. Samples were reverse-transcribed into cDNA using the QuantiTect® Reverse Transcription Kit (Qiagen). The cDNA was stored at -70°C until required for qPCR.

Two genes, *CYP6AG2* and *TPX2*, were evaluated using real-time PCR. Beacon Designer™ (Premier Biosoft) or Invitrogen’s free online primer design tool, OligoPerfect™ Designer, were used to design primers. These were based on *An. gambiae* sequence information (*CYP6AG2*: GB AY745225; *TPX2*: TIGR TC48596). The reference gene used for qPCR was based on that of Munhenga and Koekemoer [22], where studies were conducted in the laboratory on the same mosquito strains. These authors reported that *18S rRNA* showed the most stable expression of the six potential reference genes tested. The forward and reverse primer sequences for *18S* were the same as those of Munhenga and Koekemoer [22]. Each PCR reaction comprised 12.5 μl IQ™ SYBR super-mix (Bio-Rad), 4 μl primer (primer concentration optimised for each primer set), 1 μl cDNA (100 ng/μl) and nuclease-free water to a final volume of 25 μl. Cycling conditions were as follows: 93°C for 3 minutes, followed by 35 cycles of (94°C/20 seconds, ×°C/25 seconds, 72°C/30 seconds), and a final extension at 72°C for 10 minutes (followed by melt curve analysis). Primer sequences and annealing temperatures are described in Table 2. Data were analysed using the Pfaffl [27] method. For each gene of interest, the PCR product was sequenced in both directions, so that the presence of the correct product was confirmed (in addition to melt-curve analysis). Where necessary (for small amplicons i.e. < 110 base pairs), samples were cloned and then sequenced.

**Table 2:** KWAG-base/KWAG-perm primer information for qPCR (F = forward, R = reverse)

**Gene**	**Primer sequence**	**Primer concentration**	**Annealing temperature**
*CYP6AG2*	F 5′- TTG TGC TGC CGT ACT ATT CG-3′	2.0 μM	59.4°C
	R 5′- TAC TAT CGC CCG TCT CAC CT -3′		
*TPX2*	F 5′- GGA TGT TTG TGG GGA ATA CG -3′	3.5 μM	56.3°C
	R 5′- TGT GCG ATT AGC CTC CTC TT-3′		
*18S*	F 5′- TAC CTG GGC GTT CTA CTC -3′	^a^	^b^
	R 5′- CTT TGA GCA CTC TAA TTT GTT C -3′		

^a^Primer concentration was the same as that of *CYP6AG2* and *TPX4* when used as a reference gene in each case.

^b^Annealing temperature was the same as that of *CYP6AG2* and *TPX4* when used as a reference gene for each.

## Results

KWAG-base and KWAG-perm have been evaluated for insecticide resistance on an ongoing basis in the laboratory. The base strain showed very little resistance to permethrin (97.8% mean mortality 24 hours post exposure to 0.75% permethrin), while the resistant strain showed a high frequency of survivors on permethrin (42% mean mortality 24 hours post exposure to 0.75% permethrin) [22].

Microarray experiments indicated that 29 genes were over-transcribed according to the criteria outlined above. Most of these were P450 genes (55%), followed by redox genes (21%), and GSTs (14%). A group of additional genes, such as cytochrome c, a ribosomal gene (*RPS26*) and a receptor protein (*GPR npy 3*) accounted for 10% of the over-transcribed genes. The four genes with the highest transcript abundance were *CYP6AG2*, *CYP6Z1*, *TPX2* and *CYP6Z2* (Table 3), in order of statistical significance (i.e. adjusted *p*-value).

**Table 3:** List of probes over-transcribed in the resistant phenotype, KWAG-perm

**Gene**	**Function**	**Adj. *p*-value**	**FC**	**Accession number**	**Location**
*CYP6AG2*	Cytochrome P450	2.58E-7	4.1	AY745224	2R
^*#*^*CYP6Z1 (oligo)*	Cytochrome P450	3.05E-7	4.7	AF487535	3R
*TPX2*	Thioredoxin peroxidase	4.56E-7	2.3	TIGR: TC48596	3 L
^*#*^*CYP6Z2*	Cytochrome P450	1.14E-6	3.6	XM_317252	3R
*CYP6P1*	Cytochrome P450	1.14E-6	2.2	AY028785	2R
*GSTU1*	Glutathione S-transferase	3.03E-6	2.2	XM_309135	X
*SOD2*	Superoxide dismutase	3.81E-6	3.5	AY524130	2 L
*CYP12F2*	Cytochrome P450	3.81E-6	2.9	AY176050	3R
*CYP6Y2*	Cytochrome P450	3.81E-6	2.2	AY193728	3R
*GPR npy 3*	G protein coupled receptor	4.83E-6	3.2	ENSANG:	2R
				G00000009317	
*CYP9J5*	Cytochrome P450	9.14E-6	3.2	AY748830	3 L
*GPX1*	Glutathione peroxidase	9.14E-6	2.4	AY842257	2R
^*#*^*CYP6P3*	Cytochrome P450	1.68E-5	2.6	AF487534	2R
^*#*^*CYP6Z1 (cDNA)*	Cytochrome P450	1.96E-5	3.0	AF487535	3R
*CYP6M3*	Cytochrome P450	1.99E-5	4.5	AY193730	3R
*SOD1*	Superoxide dismutase	2.35E-5	2.7	AY505417	3 L
^*#*^*CYP6Z3*	Cytochrome P450	2.95E-5	3.0	AY193727	3R
*CYP6AK1*	Cytochrome P450	5.42E-5	3.6	AY745227	3 L
*CYP9M1*	Cytochrome P450	1.03E-4	2.6	AY748836	3R
*CYP12F4*	Cytochrome P450	1.68E-4	2.3	AY176048	3R
*CYP4H24*	Cytochrome P450	2.05E-4	2.6	AY062206	X
^*#*^*CYP6M2*	Cytochrome P450	3.38E-4	4.6	AY193729	3R
*GSTD1-3*	Glutathione S-transferase	6.83E-4	2.0	AF071163	2R
*CYP6M1*	Cytochrome P450	1.16E-3	2.2	AY062208	3R
*GSTS1-1*	Glutathione S-transferase	1.64E-3	2.5	L07880	3 L
*RPS26*	Ribosomal protein	2.16E-3	3.1	EMBL:	3 L
				4A3A-AAM-G-11-R	
*GPX3*	Glutathione peroxidase	2.16E-3	2.1	AY745228	X
*TPX4*	Thioredoxin-dependent peroxidase	3.60E-3	2.8	AY745235	3 L
*GSTS1-2*	Glutathione S-transferase	4.15E-3	2.3	AF513639	3 L
*Cytochrome_C*	Cytochrome c	2.17E-2	2.0	TIGR: TC48590	3R

Probes are listed in order of significance (adjusted [adj.] *p*-value), and then by fold change value. The accession number refers to Genbank, unless otherwise specified in the table; *FC* Fold change, *E* Exponent.

^#^Gene transcript validated by qPCR by Munhenga and Koekemoer [22].

**Figure 1:**
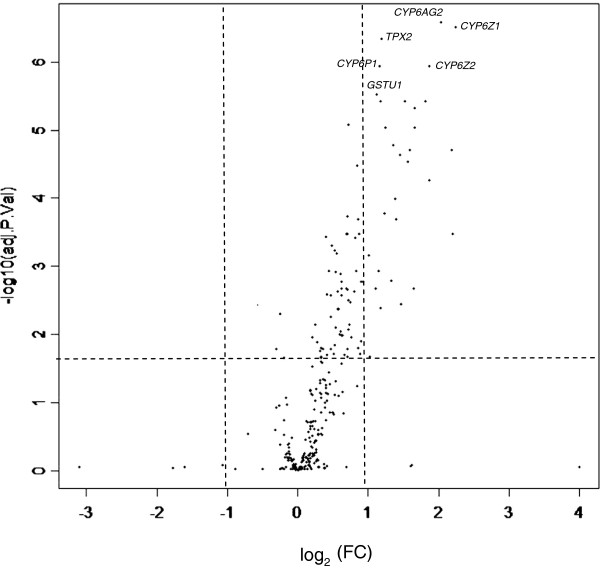
**The volcano plot of KWAG-perm versus KWAG-base microarray data.** The cut-offs for significance are indicated by the dotted lines (adj. *p*-value ≤ 0.05; FC ≥ 2) and the top 6 genes that met this criteria have been labeled. All positive FC values belong to the genes that are over-transcribed in the resistant strain (KWAG-perm), while all negative FC values belong to genes that are over-transcribed in the susceptible strain.

Two significantly over-transcribed genes, *CYP6AG2* and *TPX2*, were selected for qPCR validation of microarray data. The two other “top” genes (*CYP6Z1* and *CYP6Z2*) were already evaluated with qPCR [22]. *CYP6AG2* was the most significantly over-transcribed gene, and *TPX2* is of interest because the *TPX* genes have been found to be important in other studies reporting on insecticide resistance [8,10]. The genes *CYP6AG2* and *TPX2* produced fold change values of 2.9 and 4.5 respectively (Figure 1). The FC value of *CYP6AG2* and *TPX2* were comparable to those obtained by microarray analysis.

**Figure 2:**
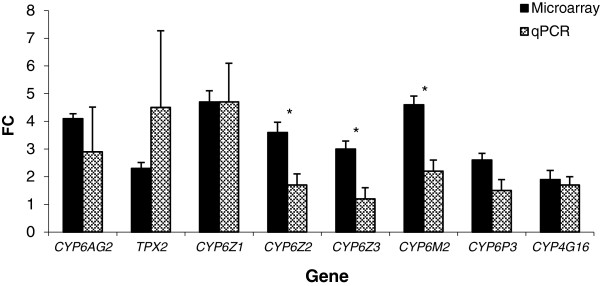
**A comparison between average fold change (FC) +SEM values recorded using microarrays and qPCR.** qPCR FC values included here for *CYP6Z1*, *CYP6Z2*, *CYP6Z3*, *CYP6M2*, *CYP6P3* and *CYP4G16* are those reported by Munhenga and Koekemoer [22]. Microarray and qPCR FC values were compared to each other by means of a *t*-test. Pairs of bars marked with a * are significantly different.

Munhenga and Koekemoer [22] used qPCR to evaluate transcription of specific genes (identified through published literature) that have been implicated in permethrin resistance in *An. arabiensis*. These included *CYP6Z1*, *CYP6Z2*, *CYP6Z3*, *CYP6M2*, *CYP6P3* and *CYP4G16*. Of these, five appeared on the list of over-transcribed genes in this microarray study (Table 3) and three of these genes [*CYP6Z1* (4.7-fold), *CYP6Z2* (1.7-fold) and *CYP6M2* (2.2-fold)] were significantly over-transcribed in the qPCR study [22]. The FC values of all six genes analysed by Munhenga and Koekemoer [22] were compared with the FC values obtained here for the same genes using microarray analysis (Figure 2). The FC values obtained by microarray analyses were compared with those obtained using qPCR for each gene using a *t*-test. The FC values of only three of the genes were significantly different to the FC values obtained by microarray analysis. These were *CYP6Z2* (*p* = 0.0251), *CYP6Z3* (*p* = 0.0219) and *CYP6M2* (*p* = 0.0092).

### Transcript profile comparison between the DDT-selected strain and the pyrethroid-selected strain (this study)

**Figure 3:**
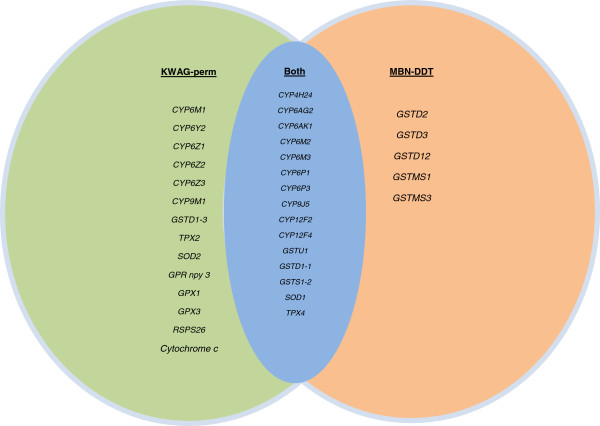
**The list of over-transcribed probes obtained in the present study was compared with that obtained in the previous study of Nardini *et al. ***[8]**.** Fifteen genes were over-transcribed in both strains. However, 14 genes were unique in the KWAG-perm strain and were not over-transcribed in the deltamethrin-resistant strain. Five of the over-transcribed genes were unique to the deltamethrin strain and all of these were GSTs.

Both MBN-DDT and KWAG-perm originate from Mamfene (KZN). However, they were placed under different insecticide selection pressures (depending on the insecticide resistance phenotypes present at the time of colonisation). The list of over-transcribed probes obtained in the permethrin-resistant strain (KWAG-perm, pyrethroid resistance only) was compared with that obtained in the DDT selected strain (MBN-DDT, pyrethroid and DDT resistance [8]) (Figure 3). In MBN-DDT, *kdr* was closely associated with DDT and permethrin resistance, while deltamethrin resistance was largely based on enhanced enzymatic detoxification. Five GST transcripts were unique in the DDT-selected strain (MBN-DDT), while 14 transcripts were unique to the permethrin-selected strain (KWAG-perm). Fifteen gene transcripts were shared between these of which 10 (66%) belonged to the P450-enzyme family.

## Discussion

The use of controlled insecticide selection in the laboratory aids in the study of changes in resistance status and its effect on certain adaptive features [28]. A list of 29 over-transcribed genes according to the criteria used (adjusted *p*-value ≤ 0.05; FC ≥ 2) was produced for the KWAG-perm strain. Most of the genes that were over-transcribed belong to the cytochrome P450 superfamily, supporting the findings of previous studies [16,22]. The four most statistically significant P450 genes included *CYP6AG2*, *CYP6Z1*, *CYP6Z2* and *CYP6P1*, all of which belong to the CYP6 family and are well-known for their role in insecticide resistance [14,29]. The most significant gene, *CYP6AG2*, is not well characterised and it would be valuable to determine the functionality of this enzyme. This gene is located on the chromosome arm 2R, a region that is not typically linked to pyrethroid resistance. Another gene in this family, *CYP6AG1*, was identified in a wild deltamethrin resistant *An. arabiensis* population from Cameroon [10]. *CYP6Z1, CYP6Z2* and *CYP6M2* showed increased transcription in this study as well as in the study by Munhenga and Koekemoer [22].

Pyrethroids are also known to cause oxidative stress by inducing lipid peroxidation, protein oxidation, and depletion of reduced glutathione [30]. This effectively increases the toxicity of the insecticide. A system of enzymes, including the superoxide dismutases (SODs), catalases, peroxidases, cytochrome C and GSTs, is present and provides defense against these reactive oxygen species (ROS) [31,32]. If ROS are not metabolised, they damage important compounds such as lipids, proteins, nucleic acids and carbohydrates, and ultimately cause cell death [32]. A number of redox enzymes showed elevated levels of transcription in KWAG-perm, which may be associated with resistance to pyrethroids. These included thioredoxin peroxidases (*TPX2* and *TPX4*), glutathione peroxidases (*GPX1* and *GPX3*), superoxide dismutases (*SOD1* and *SOD2*) and cytochrome C. Superoxide radicals are converted to hydrogen peroxide and oxygen by SODs, and hydrogen peroxide is converted to water and oxygen by catalases; or to water, by peroxidases [33].

Four GSTs (*GSTU1*, *GSTD1-3*, *GSTS1-1*, *GSTS1-2*) were also over-transcribed in KWAG-perm. Over-transcription of GSTs has been observed before in pyrethroid resistant *An. arabiensis*[10], *An. gambiae*[7,9,34] and in other insects [35-37]. In conjunction with glutathione, GSTs function as antioxidants by limiting peroxidation and by limiting (termination) “free-radical cascades” (see [30]). In addition, the GSTs are able to bind to pyrethroids and provide protection by sequestration [37].

In the recent study of Nardini *et al.*[8], the authors identified genes that were over-transcribed in a DDT and pyrethroid resistant strain (MBN-DDT). These genes were mainly found to be associated with the observed cross resistance to deltamethrin (the use of synergists clarified the roles of the over-transcribed genes, and implicated *kdr* in DDT and permethrin resistance). The gene transcripts that were identified and which are shared between the permethrin-selected (KWAG-perm) and DDT-selected strains (MBN-DDT), might explain the protection against deltamethrin in the DDT selected strain. In the process of selecting for DDT resistance it appears that there was also selection for increased transcription of genes (n = 15) that coincidentally protect against pyrethroids as well. However, the converse was not true and the pyrethroid selected strain did not develop DDT resistance. This implies that DDT selection pressure can also result in the *de novo* development of resistance to pyrethroids. Very few reports of DDT resistance without resistance to pyrethroids are available (e.g. [38]), which supports this hypothesis.

It is possible that the genes that are unique in each instance (KWAG-perm versus MBN-DDT) are more important for resistance to either type I (e.g. *GSTD2*) or type II pyrethroid (*CYPZ1*), while those that are common are likely to play a role in resistance to both kinds of pyrethroid (e.g. *CYP6M2*). Those genes that are shared between the pyrethroids and DDT selected strain might be able to metabolise both classes of insecticides (DDT as well as pyrethroids). This is specifically true for *CYP6M2*[39], but to our knowledge similar metabolic studies are not available for the other 10 genes. The role of genes conferring carbamate resistance identified in the DDT-selected strain still needs to be clarified [8]. However, P450s have been shown to provide protection against both pyrethroids and carbamates in *An. funestus*[40].

## Conclusions

The challenge with detoxification based resistant mechanisms is that it is not possible to develop a single molecular assay for the detection of resistance (as in the case of target-site mediated resistance, for example, *kdr* detection). Furthermore, in target-site resistance mechanisms, genotype usually correlates with phenotypic outcome. In the case of enzymatic resistance, this is not necessarily true. For example, three GSTs were over-transcribed in the strain here (the activity of GSTs is known to confer DDT resistance in mosquitoes [41,42]), but no DDT resistance is present in KWAG-perm. In addition, different genes were over-transcribed in the permethrin and deltamethrin resistant strains.

Microarrays are extremely useful for identifying a large number of genes that are associated with a particular resistance phenotype and in particular, transcripts that are unique to a particular phenotype. However, this technology is extremely expensive and not available in most African countries and an alternative to this approach is needed. Due to this problem, the results from the costly microarray approach used in this study were compared against those obtained by a less expensive qPCR study using the same *An. arabiensis* strain [22]. Selection of genes to be included in this qPCR study were identified through published literature [7,9,10,14,34]. This approach was used successfully by Munhenga and Koekemoer [22] who found ≥1.5 FC in transcription in four of the six genes that were selected for analysis. Five of the six genes from Munhenga and Koekemoer [22] were also identified through this microarray study. However, *CYP6AG2* and *TPX2* were novel in this study.

These data suggest that relatively inexpensive qPCR studies can successfully be used to identify increased transcription of specific metabolic genes, especially in countries that do not have access to advanced molecular systems eg. microarray analysis. However, many transcripts might be overlooked using qPCR due to the specificity of the technique. Microarray studies will in future expand the number of transcripts available for qPCR screening.

The GPIRM has initiated a “five pillar” approach against insecticide resistance. One of these pillars is to gain knowledge on mechanisms of insecticide resistance and on the effects of current insecticide resistance management strategies [1]. The present study and similar studies, contribute to achieving this goal.

## Abbreviations

CYP: Cytochrome P450; DDT: Dichlorodiphenyltrichloroethane; GPX: Glutathione peroxidase; GST: Glutathione S-transferase; kdr: Knockdown resistance; KWAG-perm: Permethrin-resistant *An. arabiensis* from South Africa; KZN: KwaZulu-Natal; LIMMA: Linear Models in Microarray Analysis; MBN-DDT: DDT and pyrethroid resistant *An. arabiensis* from South Africa; qPCR: Quantitative real-time PCR; ROS: Reactive oxygen species; SOD: Superoxide dismutase; TPX: Thioredoxin peroxidase; WHO: World Health Organization.

## Competing interests

The authors declare that they have no competing interests.

## Authors’ contributions

LN performed all the experimental work, analyses and drafted the MS. RC provided technical support for the microarray work and reviewed the MS. NC assisted with the microarray data analysis and reviewed the MS. LK conceived the project, provided funding and reviewed the MS. All authors read and approved the final manuscript.
